# *Akkermansia muciniphila* in patients with metabolic dysfunction-associated steatotic liver disease

**DOI:** 10.25122/jml-2024-0342

**Published:** 2024-09

**Authors:** Adina Ioana Mihele, Liviu Lazar

**Affiliations:** 1 Doctoral School of Biomedical Sciences, Faculty of Medicine and Pharmacy, University of Oradea, Oradea, Romania; 2 Department of Medical Disciplines, Faculty of Medicine and Pharmacy, University of Oradea, Oradea, Romania; 3 Department of Psycho-Neuroscience and Recovery, Faculty of Medicine and Pharmacy, University of Oradea, Oradea, Romania

**Keywords:** MASLD, *Akkermansia muciniphila*, correlation, liver markers

## Abstract

*Akkermansia muciniphila* (AM), one of the many microbial species residing in the human gut, has been particularly highlighted for its potential beneficial impacts on host metabolism and gut barrier function. This study evaluated the association between AM concentration and metabolic markers among patients diagnosed with metabolic dysfunction-associated steatotic liver disease (MASL). The study included a cohort of 122 patients with MASLD, monitored between January 1 and June 30, 2024, at the Venus Vascular Center in Oradea, Romania. Enterotype 2 was predominant in the study population, accounting for over 60% of participants. Correlation analysis revealed no statistically significant association between alanine aminotransferase (ALT) or aspartate aminotransferase (AST) levels and AM concentration (ALT: r = -0.147, *P* = 0.105; AST: r = -0.090, *P* = 0.325). However, a significant negative linear correlation was determined between gamma-glutamyl transferase (GGT) values and AM concentrations (r = -0.314, *P* < 0.001) and a moderate, positive correlation between high-density lipoprotein (HDL) values and AM concentration (r = 0.307, *P* < 0.001). Glycemia showed a weak negative correlation with AM concentration (r = -0.262, *P* = 0.003). The improvement of liver markers (AST, ALT), even in the absence of correlation with AM concentration, and the negative correlation of GGT, a marker for hepatobiliary diseases and metabolic syndrome, suggest the reduction of oxidative stress in MASLD.

## INTRODUCTION

In recent years, the gut microbiota has attracted significant attention as a determinant of metabolic health, controlling numerous physiological processes. One of many microbial species living in the human gut, *Akkermansia muciniphila* (AM), has been particularly highlighted for its potential beneficial impacts on host metabolism and gut barrier [1]. Initially discovered for its mucin-degrading capabilities, AM has now been linked with multiple metabolic diseases, including obesity, type 2 diabetes, and non-alcoholic fatty liver disease (NAFLD) — now more accurately described as metabolic dysfunction-associated steatotic liver disease (MASLD) [2].

MASLD is characterized by elevated liver fat levels and is often associated with insulin resistance, dyslipidemia, and chronic inflammation, which can progress to more severe forms of the disease. These include metabolic dysfunction-associated steatohepatitis (MASH) and metabolic dysfunction-associated steatotic liver (MASL). Several bacterial species, including *Bacteroides fragilis, Escherichia coli*, and *Helicobacter pylori*, are known to promote the development of MASL by compromising the intestinal barrier, triggering pro-inflammatory cytokine production, and facilitating immune cell infiltration into the liver, which contributes to liver dysfunction. In contrast, species like *Akkermansia muciniphila* and *Bifidobacterium* exert protective effects by supporting the intestinal barrier and producing anti-inflammatory metabolites, such as short-chain fatty acids [3].

A complex relationship between AM and metabolic markers in MASL and its complications emerges [3]. Numerous studies have found that increased levels of AM correlate with improved insulin sensitivity, less inflammation, and a better lipid profile. Conversely, lower levels of AM have been linked to metabolic dysfunction and an increased prevalence of metabolic syndrome components, including hyperglycemia, dyslipidemia, and hepatic steatosis [4].

With MASL being so prevalent globally and associated with significant morbidity/mortality, it is imperative to investigate microbial drivers of disease development. The gut-liver axis is a bidirectional communication pathway between the gut microbiota and the liver, which has been seen as one of the essential effectors in MASL pathogenesis [5]. In this context, AM has emerged as a key regulator of gut integrity and a modulator of various metabolic processes.

This study evaluated the association between AM concentration and metabolic markers among patients diagnosed with MASL. Identifying the critical correlation of the gut microbiota in MASL, specifically the implications of AM as a potential biomarker for disease progression, may offer new specific treatment targets for this increasingly prevalent pathology.

## Material and Methods

### Study design

This study was conducted on a cohort of patients diagnosed with MASL, monitored from January 1 to June 30, 2024, at the Venus Vascular Center in Oradea, Romania. The diagnosis of MASL followed the Clinical Practice Guidelines for managing MASL, established by the European Association for the Study of the Liver, the European Association for the Study of Diabetes, and the European Association for the Study of Obesity [6].

Inclusion criteria

- Adults (≥18 years old)
- Confirmed diagnosis of MASL

Exclusion criteria

- Children, pregnancy, breastfeeding
- Medication affecting the digestive tract within the last 6 months
- Use of corticosteroids, amiodarone, lomitapide, or valproate within the last 6 months
- Recent invasive or therapeutic procedures on the digestive tract (within the last 6 months)
- Active upper/lower gastrointestinal or genital bleeding
- Alcohol consumption >20 g/day for women, >30 g/day for men
- Positive tests for hepatitis viruses (A, B, C, D, E), cytomegalovirus, Epstein-Barr virus, cirrhosis
- Cancer
- Elevated liver enzymes
- Allergies

### Data collection

The collection of clinical and laboratory data involved several steps:

1. Each participant was screened according to the inclusion and exclusion criteria (based on medical history, physical examination, and abdominal ultrasound).
2. Participants provided a stool sample using a sterile collection kit, which was then transported to the center’s laboratory and sent to a private laboratory for processing. DNA identification was used to analyze the intestinal microbiota species.
3. On the same day, after a fasting period of at least 8 hours, venous blood samples were collected in standardized vacutainers to determine the values of the following biochemical parameters: aspartate aminotransferase (AST; reference range 0–35 U/L), alanine aminotransferase (ALT; reference range 0–45 U/L), triglycerides (TG; reference range 50–200 mg/dL), gamma-glutamyl transferase (GGT; reference range 0–49 U/L), total bilirubin (TB; reference range 0.1–1.1 mg/dL), total cholesterol (TC; reference range 70–200 mg/dL), high-density lipoprotein (HDL; reference range 35–65 mg/dL), low-density lipoprotein (LDL; reference range 0–150 mg/dL), and serum glucose (reference range 70–105 mg/dL).

### Statistical analysis

Data were analyzed using IBM SPSS Statistics software [20]. Univariate dispersion analysis (Levene's Test) was used to check the homogeneity of variances among the different groups. If the data were homogeneous, parametric tests (ANOVA) were subsequently used. The graphs were created and edited using various programs, including SPSS, Microsoft Excel (version 2021), Matplotlib (version 3.7.1), and Seaborn (version 0.12.2) in Python. A statistical difference (*P*) was considered significant if its value was less than 0.05.

## RESULTS

**Figure 1:**
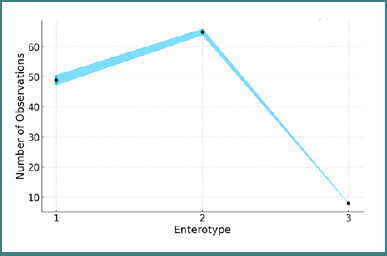
Incidence of enterotypes in the study group

According to the inclusion criteria, 130 subjects were initially included in the study. After applying the exclusion criteria, 122 participants completed the study. Three participants were excluded due to recent antibiotic use, three for taking proton pump inhibitors, and two for elevated serum transaminase levels. The clinical and laboratory characteristics of the study subjects are presented in Table 1. The majority of subjects had enterotype 2, followed by enterotypes 1 and 3, as illustrated in Figure 1.

**Table 1:** Clinical and laboratory data of participants

Parameter	Study group
**DD**	
Age, years, mean ± SD (min, max)	54.16 ± 12.17 (25, 74)
Male gender, n (%)	75 (61.48)
Urban residence, n (%)	85 (69.67)
**Clinical data**	
Co-morbidities, n (%)	50 (40.98)
hypertension	10 (8.20)
diabetes	35 (20.49)
dyslipidemia	89 (72.95)
obesity	56 (45.90)
**Paraclinical investigations, mean ± SD**	
ALT, (U/L)	30.07 ± 15.11
AST, (U/L)	25.63 ± 8.36
TB, (mg/dL)	0.6 ± 0.21
GGT, (U/L)	55.90 ± 38.70
TC, mg/dL	203.35 ± 62.36
HDL, mg/dL	43.06 ± 13.51
LDL, mg/dL	118.96 ± 49.27
TG, mg/dL	195.16 ± 121.17
	
Serum glucose, mg/dL	114.04 ± 31.33

DD, demographic data; ALT, alanine aminotransferase; AST, aspartate aminotransferase; TG, triglycerides; GGT, gamma-glutamyl transferase; TB, total bilirubin; TC, total cholesterol; HDL, high-density lipoprotein; LDL, low-density lipoprotein; n, number; M, mean; SD, standard deviation; min, minimum; max, maximum.

**Figure 2:**
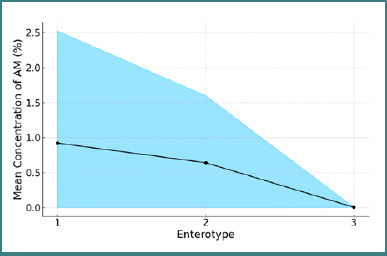
Variation in the percentage concentration of *Akkermansia muciniphila* across different enterotypes

The homogeneity of variances in the percentage concentration of AM across different enterotypes was tested using Levene's test. The test results support the homogeneity of the data (Levene's statistic = 2.13, *P* = 0.123). Subsequently, the variations among the data were examined using ANOVA analysis. Although a decreasing trend in the percentage concentration of AM among the enterotypes was identified (0.923% ± 1.601% for enterotype 1, 0.641% ± 0.959% for enterotype 2, and 0.007% ± 0.0009% for enterotype 3), the one-way ANOVA analysis did not support a statistically significant difference among the different enterotypes [F(2, 119) = 2.08, *P* = 0.129], as shown in Figure 2.

**Figure 3:**
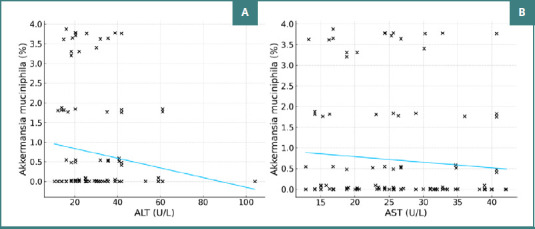
Correlation between ALT, AST, and the percentage concentration of AM. A, Correlation between ALT (U/L) and the percentage concentration of AM; B, Correlation between AST (U/L) and the percentage concentration of AM

No significant linear relationship was observed between ALT and AST values and AM concentration in the study group (ALT: r = -0.147, *P* = 0.105; AST: r = -0.090, *P* = 0.325) or monotonic relationship (ALT: ρ = -0.121, *P* = 0.183; AST: ρ = -0.120, *P* = 0.188), as shown in Figure 3AB.

**Figure 4:**
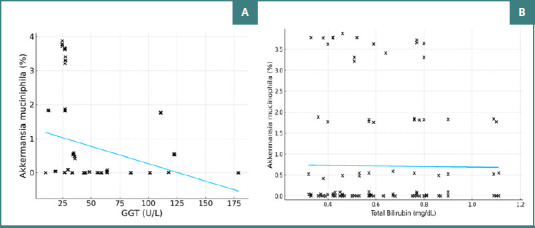
Correlation between GGT, TB, and the percentage concentration of AM. A, Correlation between GGT (U/L) and the percentage concentration of AM; B, Correlation between TB (mg/dL) and the percentage concentration of AM.

A statistically significant negative linear correlation (r = -0.314, *P* < 0.001) was observed between GGT values and the percentage concentrations of AM in the study group, indicating a modest decrease in AM concentration as GGT levels increase (slope = -0.0102, R^2^ = 0.098; Figure 4A). No statistically significant correlation was found between TB and AM in the study group (r = -0.012, *P* = 0.898; Figure 4B).

**Figure 5:**
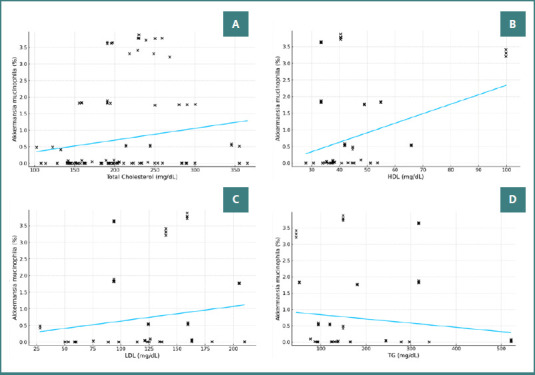
Correlation between lipid profile markers and the percentage concentration of Akkermansia muciniphila. A, Correlation between TC (mg/dL) and the percentage concentration of AM. B, HDL (mg/dL); C, LDL (mg/dL); D, TG (mg/dL).

For total cholesterol (TC), no significant correlation with AM concentration was observed (r = 0.176, *P* = 0.052; Figure 5A). However, a moderate, positive, statistically significant correlation was identified between HDL values and AM concentration (r = 0.307, *P* < 0.001), with a slope of 0.0286 and an R^2^ value of 0.0944 (Figure 5B). No significant correlation was found between AM concentration and LDL (r = 0.172, *P* = 0.057) or triglycerides (r = -0.125, *P* = 0.170), as presented in Figure 5CD.

**Figure 6:**
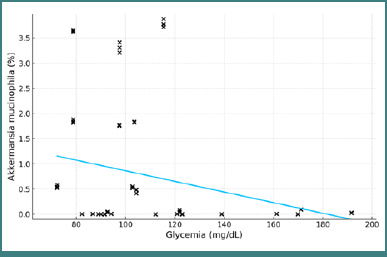
Correlation between blood glucose and the percentage concentration of AM

Blood glucose was weakly but significantly negatively correlated with AM concentration (r = -0.262, *P* = 0.003), with a slope of -0.0105 and an R^2^ value of 0.0688 (Figure 6).

## Discussion

MASLD affects approximately 25% of the global population, and its prevalence continues to rise, threatening to become a global health issue [7]. MASLD is closely linked to metabolic syndrome and can progress to more severe conditions, such as fibrosis, liver cirrhosis, or liver cancer. The progression of this disease is strongly influenced by the composition and function of the gut microbiota via the 'gut-liver axis' [7,8].

The discovery of enterotypes has been pivotal in understanding the relationship between gut microbiota and metabolism. The human gut contains over 1,000 bacterial species, with *Firmicutes, Bacteroidetes, Actinobacteria*, and *Proteobacteria* playing key roles in metabolic processes [9]. Classifying the gut microbiota into three distinct enterotypes holds promise for personalized medicine, particularly in restoring microbial balance as a potential therapeutic strategy.

The restoration of gut microbiota balance could emerge as a significant treatment direction. Gut microbiota alterations have been implicated in the development of MASLD, with changes such as the overgrowth of ethanol-producing *Escherichia coli* identified as contributing factors [10]. When the inflammasome is deficient, the liver becomes exposed to endotoxins, leading to inflammation and liver damage [11,12]. The study aimed to identify the predominant enterotypes in patients with MASL and their correlation with different liver and metabolic markers.

In our study group, enterotype 2 was predominant (more than 60%). The systematic review published by Heng Yuan *et al*. [11] showed a similar incidence of *Prevotella* enterotype (enterotype 2) in Caucasian patients (65.1%) with metabolic dysfunction-associated fatty liver disease and a very high incidence in Asian patients (more than 90%). The *Prevotella copri* subtype, which impacts *Bifidobacterium* levels, is thought to contribute to the dysbiosis associated with fatty liver disease [11]. The incidence of MASLD was correlated with enterotype, age, obesity, and ethnicity (*P* < 0.05). Asian patients with MASLD showed decreased *Firmicutes*, AM, and increased *Bacteroidetes* and *Prevotella copri* [11].

AM is an intestinal anaerobic bacterium recognized for its beneficial effects on metabolic dysfunction in fatty liver disease and is considered to have probiotic properties. It may be considered a marker of health [13,14]. One of its primary roles is the degradation of mucin in the gut lining. Although research has shown that AM is not specifically associated with any particular enterotype [15], a low abundance of AM has been consistently linked to MASLD in animal and human studies. Its anti-inflammatory mechanisms have also been highlighted, with studies showing that AM reduces cytokine levels in Kupffer cells and suppresses macrophage activity, helping to modulate liver inflammation [6,16]. In this study, we observed a decreasing trend in AM concentration across different enterotypes, though the differences were not statistically significant. This reduction in AM is consistent with the known association between MASLD and obesity, as reported in previous studies [17]. Heng Yuan *et al*. [11] also observed reduced AM levels in Asian patients with MASLD. Moreover, the abundance of AM shows an inverse correlation with metastatic diseases in general [18].

AST, ALT, and GGT levels were determined to assess the degree of liver injury among the patients included in the study. Studies support the role of AM as a protector against liver disease. Administration of AM reduces hepatotoxicity and serum transaminase levels [18]. In a study by Clara Depommier *et al*. (2019), AM administration to humans (*n* = 40) led to a reduction in inflammatory and hepatic markers, including GGT, AST, and ALT, without disrupting the overall microbiota composition [17]. Similar results were found in rodent models, where AM administration significantly reduced AST, ALT, and triglyceride levels and partially restored bacterial diversity [19]. Our study revealed a significant negative linear correlation (r = -0.314, *P* < 0.001) between GGT values and AM concentrations. This finding supports the potential of AM to reduce liver markers as its abundance increases, suggesting a promising avenue for further research and potential health benefits.

A moderate, positive correlation was identified between HDL and AM concentration. No statistically significant correlation was identified between AM concentration and LDL and TG. Studies in rodents have been published that support the effect of AM on cardiovascular diseases; specifically, the administration of AM leads to a decrease in total cholesterol, LDL-cholesterol, and TG, reducing atherosclerosis [18].

In the current study, glycemia showed a weak but statistically significant negative correlation with AM concentration. A review by Niu *et al*. presents both supporting and conflicting evidence regarding these findings [20]. For example, a study by Zhang *et al*. [21] argues that AM abundance is lower in patients with pre-diabetes and diabetes. Régnier *et al*. [22] survey showed that after diabetes induction in mice, AM abundance was higher in those without diabetes. Additionally, AM has been shown to potentially reduce glucose depletion, further supporting its role in glucose regulation [23].

## Conclusion

The improvement of liver markers (AST, ALT), even in the absence of correlation with the percentage concentrations of AM, and the negative correlation of GGT, a marker for hepatobiliary diseases and metabolic syndrome, suggest the reduction of oxidative stress in MASLD. The study supports the association of AM abundance with serum HDL, LDL, and glucose levels. Further microbiota studies are needed on the abundance of this type of bacteria and how it interacts with microbiota-specific bacteria.

## Personal thanks

The authors sincerely thank the University of Oradea for providing the logistical support and facilities that made this study possible.

## References

[1] Jian H, Liu Y, Wang X, Dong X, Zou X (2023). *Akkermansia muciniphila* as a Next-Generation Probiotic in Modulating Human Metabolic Homeostasis and Disease Progression: A Role Mediated by Gut-Liver-Brain Axes?. Int J Mol Sci.

[2] Cani PD, de Vos WM (2017). Next-Generation Beneficial Microbes: The Case of *Akkermansia muciniphila*. Front Microbiol.

[3] Saenz E, Espinosa Montagut N, Wang B, Stein-Thöringer C, Wang K, Weng H (2024). Manipulating the Gut Microbiome to Alleviate Steatotic Liver Disease: Current Progress and Challenges. Engineering.

[4] Plovier H, Everard A, Druart C, Depommier C, Van Hul M, Geurts L (2017). A purified membrane protein from Akkermansia muciniphila or the pasteurized bacterium improves metabolism in obese and diabetic mice. Nat Med.

[5] Depommier C, Everard A, Druart C, Plovier H, Van Hul M, Vieira-Silva S (2019). Supplementation with Akkermansia muciniphila in overweight and obese human volunteers: a proof-of-concept exploratory study. Nat Med.

[6] European Association for the Study of the Liver (EASL), Electronic address: easloffice@easloffice.eu; European Association for the Study of Diabetes (EASD); European Association for the Study of Obesity (EASO); European Association for the Study of the Liver (EASL) (2024). EASL-EASD-EASO Clinical Practice Guidelines on the management of metabolic dysfunction-associated steatotic liver disease (MASLD). J Hepatol.

[7] Albillos A, de Gottardi A, Rescigno M (2020). The gut-liver axis in liver disease: Pathophysiological basis for therapy. J Hepatol.

[8] Jadhav PA, Thomas AB, Nanda RK (2024). Correlation of non-alcoholic fatty liver disease and gut microflora: clinical reports and treatment options. Egypt Liver J.

[9] Mokhtari Z, Gibson DL, Hekmatdoost A (2017). Nonalcoholic Fatty Liver Disease, the Gut Microbiome, and Diet. Adv Nutr.

[10] Zhu L, Baker RD, Baker SS (2015). Gut microbiome and nonalcoholic fatty liver diseases. Pediatr Res.

[11] Yuan H, Wu X, Wang X, Zhou JY, Park S (2024). Microbial Dysbiosis Linked to Metabolic Dysfunction-Associated Fatty Liver Disease in Asians: *Prevotella copri* Promotes Lipopolysaccharide Biosynthesis and Network Instability in the Prevotella Enterotype. Int J Mol Sci.

[12] Henao-Mejia J, Elinav E, Jin C, Hao L, Mehal WZ, Strowig T (2012). Inflammasome-mediated dysbiosis regulates progression of NAFLD and obesity. Nature.

[13] Rao Y, Kuang Z, Li C, Guo S, Xu Y, Zhao D (2021). Gut Akkermansia muciniphila ameliorates metabolic dysfunction-associated fatty liver disease by regulating the metabolism of L-aspartate via gut-liver axis. Gut Microbes.

[14] Ghotaslou R, Nabizadeh E, Memar MY, Law WMH, Ozma MA, Abdi M (2023). The metabolic, protective, and immune functions of Akkermansia muciniphila. Microbiol Res.

[15] Derrien M, Belzer C, de Vos WM (2017). Akkermansia muciniphila and its role in regulating host functions. Microb Pathog.

[16] Han Y, Li L, Wang B (2022). Role of Akkermansia muciniphila in the development of nonalcoholic fatty liver disease: current knowledge and perspectives. Front Med.

[17] Depommier C, Everard A, Druart C, Maiter D, Thissen JP, Loumaye A (2021). Serum metabolite profiling yields insights into health promoting effect of A. muciniphila in human volunteers with a metabolic syndrome. Gut Microbes.

[18] Xia J, Lv L, Liu B, Wang S, Zhang S, Wu Z (2022). Akkermansia muciniphila Ameliorates Acetaminophen-Induced Liver Injury by Regulating Gut Microbial Composition and Metabolism. Microbiol Spectr.

[19] Kim S, Lee Y, Kim Y, Seo Y, Lee H, Ha J (2020). Akkermansia muciniphila Prevents Fatty Liver Disease, Decreases Serum Triglycerides, and Maintains Gut Homeostasis. Appl Environ Microbiol.

[20] Niu H, Zhou M, Zogona D, Xing Z, Wu T, Chen R (2024). *Akkermansia muciniphila:* a potential candidate for ameliorating metabolic diseases. Front Immunol.

[21] Zhang X, Shen D, Fang Z, Jie Z, Qiu X, Zhang C (2013). Human gut microbiota changes reveal the progression of glucose intolerance. PLoS One.

[22] Régnier M, Rastelli M, Morissette A, Suriano F, Le Roy T, Pilon G (2020). Rhubarb Supplementation Prevents Diet-Induced Obesity and Diabetes in Association with Increased *Akkermansia muciniphila* in Mice. Nutrients.

[23] Everard A, Belzer C, Geurts L, Ouwerkerk JP, Druart C, Bindels LB (2013). Cross-talk between Akkermansia muciniphila and intestinal epithelium controls diet-induced obesity. Proc Natl Acad Sci U S A.

